# Brodifacoum pharmacokinetics in acute human poisoning: implications for estimating duration of vitamin K therapy

**DOI:** 10.1080/24734306.2021.1887637

**Published:** 2021-03-01

**Authors:** Daniel G. Nosal, Richard B. van Breemen, John W. Haffner, Israel Rubinstein, Douglas L. Feinstein

**Affiliations:** a Linus Pauling Institute, Oregon State University, Corvallis, OR, USA;; b Department of Emergency Medicine, University of Illinois, Peoria, IL, USA;; c Department of Medicine, University of Illinois, Chicago, IL, USA;; d Jesse Brown VA Medical Center, Chicago, IL, USA;; e Department of Anesthesiology, University of Illinois, Chicago, IL, USA

**Keywords:** Brodifacoum, synthetic cannabinoids, rodenticide, vitamin K1

## Abstract

Standard of care follow-up therapy for patients poisoned by long-acting anticoagulant rodenticides (LAARs) is daily high-dose (up to 100 mg per day) oral vitamin K1 (VK_1_) for weeks to months to over a year. The availability of CLIA-certified quantitative testing for plasma LAAR concentrations can now assist health care providers in determining when to safely discontinue VK_1_ therapy. We present estimates of treatment duration required to reach safe concentrations (< =10ng/ml) using serial measurements of plasma brodifacoum (BDF, a potent LAAR) concentrations obtained from patients poisoned after inhaling synthetic cannabinoids containing BDF. We fit the data to zero-order (linear) and first-order (exponential) curves, the latter to account for enterohepatic circulation of BDF. The results show that estimates of therapy duration are significantly longer when exponential clearance is assumed. Accordingly, we recommend that plasma BDF concentrations be monitored simultaneously with international normalization ratio (INR) during follow-up of poisoned patients, and that concentrations be determined after VK_1_ therapy is discontinued to document persistence of safe concentrations.

## Introduction

A recent outbreak of long-acting anticoagulant rodenticide (LAAR)-associated coagulopathy and life-threatening bleeding in Illinois and several other States [1–3] focused attention on long-term treatment of this condition with vitamin K1 (VK_1_). Current standard of care treatment of patients poisoned by the LAAR brodifacoum (BDF) is VK_1_ given intravenously or orally, thereafter requiring supplementation for durations of weeks to months. Extended treatment is due to the long biological half-life of BDF, resulting from a combination of insignificant liver metabolism, lack of renal excretion, accumulation in lipophilic tissues, and enterohepatic circulation [4]. Since VK_1_ does not increase BDF clearance, treatment is required until gradual elimination leads to a safe serum level of <10 ng/ml [5]. Premature discontinuation of VK_1_ before this threshold is reached may lead to recurrence of coagulopathy and bleeding. Hence, both international normalized ratio (INR) and plasma BDF concentrations must be monitored during follow-up. One means of estimating the duration of VK_1_ treatment is to carry out serial measurements of plasma BDF concentrations, then fitting the data to pharmacokinetic models of BDF clearance which can be described as either 1- or 2-compartment models, the latter to account for biphasic elimination and enterohepatic circulation [6]. The purpose of this study was to estimate the time required for VK_1_ treatment after acute BDF poisoning by fitting quantitative data of plasma BDF concentrations obtained from patients acutely poisoned in Peoria, Illinois.

## Methods

We previously quantified plasma BDF concentrations using a high pressure liquid chromatography-mass spectrophotometric method in blood samples obtained from patients poisoned due to inhalation of synthetic cannabinoids contaminated with BDF [7]. For this study, ‘initial BDF concentration’ refers to the concentration measured in the first blood sample collected after a patient was admitted to the emergency department; however, the time between BDF exposure to admission is not known. CurveExpert Professional v2.7.3 (Hyams Development, Chattanooga, TN) was used to directly fit plasma BDF values to zero-order (linear) or first-order (exponential) decay without linearization of the data, and we used the resulting equations to calculate the time needed to reach 10 ng/ml. Zero versus first-order derived times were compared by paired t-test, and associations by Pearson correlation analysis.

## Results

**Figure 1.**
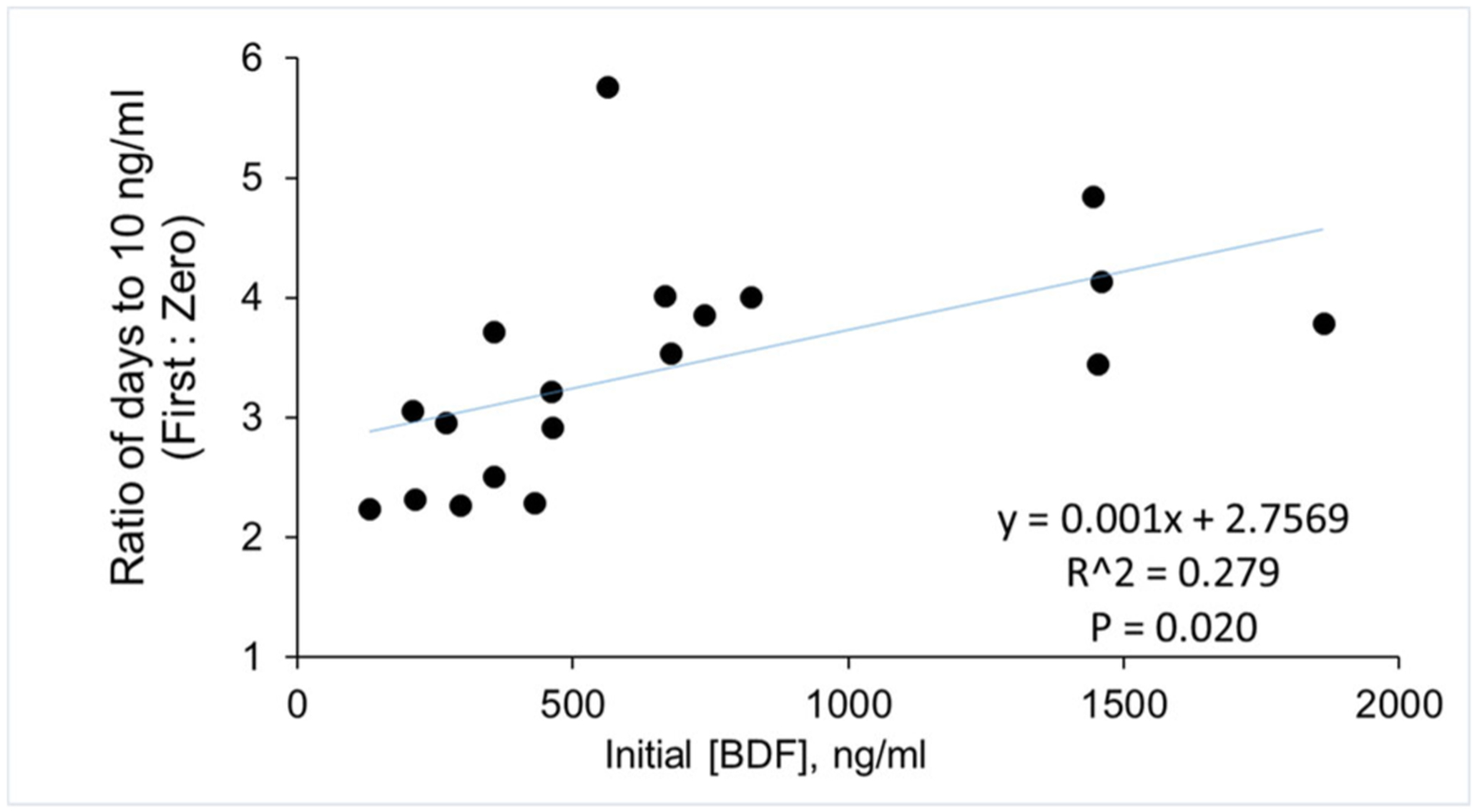
Correlation of calculated VK1 therapy times to plasma BDF concentrations. The ratio of zero-order to first-order calculated times required to reach 10 ng/ml are plotted versus the plasma BDF level measured in the first available sample for each case. P = 0.020, Pearson correlation analysis.

Of the 33 patients whose samples were available, there were 19 for whom 3 or more serial blood samples were collected. When we fit the data directly to zero-order (linear) decline, the average time to reach 10 ng/ml was 12.2 ± 8.3 days (Table 1). In contrast, in all cases the time to reach 10 ng/ml was longer when the data were fitted by exponential regression to a first-order decay (43.4 ± 35.4 days; *p* < 0.0001 versus zero-order, paired t-test). Although R-squared values for zero and first-order regressions were similar (0.622 versus 0.645, Table 1), they were significantly higher (*p* < 0.01) when first-order regression was used. We did not detect any significant associations between initial BDF concentrations measured and either the zero or first-order calculated times to 10 ng/ml. However, the values determined by zero-order regression were more similar to first-order values at lower compared to higher initial BDF concentrations, and the ratio (first-order: zero-order) was positively correlated to the initial BDF concentrations (*p* = 0.020) (Figure 1).

## Discussion

Clearance of BDF is complex due to distribution into multiple compartments, minimal metabolism, and enterohepatic circulation. While most studies have used first-order, exponential modeling to describe its pharmacokinetics [7–9], several have also used linear modeling [5, 10]. Measurements of plasma BDF concentrations made at early times relative to the initial exposure, as done here, will reflect a combination of partitioning into multiple compartments, enterohepatic circulation, and elimination. In contrast measurements done at later times when the distribution phase is complete will primarily reflect the elimination phase and ongoing enterohepatic circulation. In this study, the times between initial exposure to BDF and the first blood draw (the initial measurement) were not known, therefore samples with low initial BDF concentrations could reflect longer duration since exposure; or exposure to lower amounts. Our finding that zero-order fitting more closely paralleled first-order fitting at lower initial BDF concentrations suggest that the initial distribution phase was already completed in those cases, although it remains possible that the distribution phase at low BDF concentrations may, in some patients, follow linear kinetics.

Determining when to discontinue VK1 therapy for BDF poisoned patients is critical to avoid recurrence of life-threatening bleeding. Although INR values can indicate return to normal clotting function, that may not reflect safe plasma BDF concentrations. In our review of 21 case reports of LAAR poisoning [11], about half the patients had plasma BDF concentrations of < =10 ng/ml at discharge from hospital, presumably INRs were within normal range at discharge. More recently, in our analysis of poisonings due to inhaling synthetic cannabinoids laced with BDF [7], we found that in most cases (24 of 27 cases) although INRs had reached normal values (< =1.7) at discharge, the average plasma BDF concentration at discharge was 454 ng/ml, more than 40 times the considered safe level. In Wisconsin [12], a cohort of 19 patients also poisoned by using contaminated synthetic cannabinoids was followed for up to 102 days; and of those BDF rebound was observed in 5 cases. Whether due to patient non-adherence to VK1 treatment, repeated use of BDF-tainted cannabinoids, or if VK1 therapy was discontinued prematurely when INRs were normalized but BDF was still present leading to recurrence of anti-coagulation is not known.

## Conclusions

We strongly endorse recommendations to perform serial measurements of plasma BDF to guide the post-exposure duration of oral VK_1_ therapy in poisoned patients [5]. Adequate modeling requires multiple (at least 3) sampling of plasma concentrations be done; and based on the reported half life for BDF (7.5 ± 1.3 days, mean ± se, [7]), we recommend those be collected over a period of 1 week. Using first-order modeling to describe its pharmacokinetics can provide an estimate of the required duration of continuous VK_1_ treatment. Alternatively, assuming zero-order decline can provide an estimate when the current phase of clearance will be complete. We further recommend that at least one additional measurement of plasma BDF be carried out at the end of treatment to ensure that concentrations of 10 ng/ml or less are reached, and that a second measurement be made within 2 weeks after cessation of VK_1_ to ascertain maintenance of safe concentrations. The availability of certified laboratories in the USA to quantify plasma BDF concentration now enables such testing to be ordered by healthcare providers at point of care thereby improving patient care [5].

## Figures and Tables

**Table 1.** Calculation of time to reach safe plasma BDF concentrations.

			Days to reach 10 ng/ml			
Case #	First BDF [ng/ml]	T_1/2_ (days)	Zero Order	Ro2	First Order	Ro2
29	130	7.6	14.1	0.837	31.1	0.832
20	207	8.9	13.0	0.408	38.8	0.415
27	211	4.4	7.1	0.795	17.0	0.844
24	270	2.6	4.0	0.788	11.8	0.810
23	294	4.6	8.7	0.969	20.8	0.987
4	355	2.0	3.5	0.829	9.1	0.892
28	355	17.8	26.7	0.218	100.0	0.209
19	431	2.3	2.9	0.813	6.8	0.903
21	460	12.4	17.0	0.171	54.8	0.187
31	463	3.9	7.4	0.989	21.6	0.999
22	563	7.1	10.8	0.189	39.8	0.190
30	666	18.6	28.0	0.163	112.8	0.167
33	678	6.6	9.9	0.454	35.4	0.486
10	739	8.7	15.7	0.278	60.2	0.275
32	822	17.6	26.6	0.652	104.8	0.669
14	1443	14.0	21.0	1.000	100.4	0.999
18	1451	3.1	5.6	0.833	19.4	0.893
15	1458	3.4	5.8	0.578	24.1	0.576
26	1862	2.5	4.2	0.859	16.0	0.913
	**average**	7.8	12.2	0.622	43.4^a^	0.645^b^
	**SD**	5.6	8.3	0.302	35.4	0.315

a *p* < 0.0001 versus zero-order, paired t-test.

b *p* < 0.01 versus zero-order, paired t-test. First BDF, BDF level measured in the first blood sample collected after patient was admitted to the emergency department. T1/2, half-life determined for BDF. Data adapted from [7].
